# Infectious Etiologies of Parkinsonism: Pathomechanisms and Clinical Implications

**DOI:** 10.3389/fneur.2019.00652

**Published:** 2019-06-19

**Authors:** Nattakarn Limphaibool, Piotr Iwanowski, Marte Johanne Veilemand Holstad, Dominik Kobylarek, Wojciech Kozubski

**Affiliations:** Department of Neurology, Poznan University of Medical Sciences, Poznan, Poland

**Keywords:** encephalitis lethargica, infectious diseases, Parkinson's disease, neurodegeneration, neuroinflammation

## Abstract

Extensive research in recent decades has expanded our insights into the pathogenesis of Parkinson's disease (PD), though the underlying cause remains incompletely understood. Neuroinflammation have become a point of interest in the interconnecting areas of neurodegeneration and infectious diseases. The hypothesis concerning an infectious origin in PD stems from the observation of Parkinson-like symptoms in individuals infected with the influenza virus who then developed encephalitis lethargica. The implications of infectious pathogens have later been studied in neuronal pathways leading to the development of Parkinsonism and PD, through both a direct association and through synergistic effects of infectious pathogens in inducing neuroinflammation. This review explores the relationship between important infectious pathogens and Parkinsonism, including symptoms of Parkinsonism following infectious etiologies, infectious contributions to neuroinflammation and neurodegenerative processes associated with Parkinsonism, and the epidemiologic correlations between infectious pathogens and idiopathic PD.

## Background

Parkinson's disease (PD) is a debilitating neurodegenerative disorder manifesting as reduced facilitation of voluntary movements. It affects 1% of the population above the age of 60 years, with an annual incidence of 15 per 100,000 (1). A substantial growth in the prevalence of PD worldwide is predicted as a result of population aging and increases in life expectancy (2). Extensive research over the past few decades, including epidemiologic and genetic studies and post-mortem analysis, has expanded our insights into the pathogenesis of the disease. For the vast majority of cases, the underlying cause of PD remains incompletely understood.

It has been suggested that PD's complex and multifactorial etiology results from environmental contributions in genetically predisposed individuals (3). Genetic links have been identified by recent genome-wide association studies as causes or risk factors for PD development (4, 5). However, the sporadic nature of the occurrences suggests interactions between additional factors that has largely remained enigmatic.

Parkinsonian symptoms refer to PD-like manifestations, such as rapidly progressing rigidity, bradykinesia, postural instability, cognitive, and oculomotor abnormalities, but which do not lead to a firm diagnosis of PD. The role of bacterial and viral infections in the etiology of Parkinsonism and idiopathic PD has been indicated by recent studies, although a clear correlation is yet to be established. Parkinsonism arising from the loss of dopaminergic neurons as a consequence of an infectious process occurs rapidly, contrary to the late-onset and progressive course of idiopathic PD (6). Although infection-dependent Parkinsonism and idiopathic PD are distinct entities, the role of infectious pathogens have been implicated in both pathologies whether in the disease pathogenesis or through epidemiologic correlations.

The original hypothesis of an infectious origin in PD stems from the observation of Parkinson-like symptoms in individuals infected with the influenza virus who developed encephalitis lethargica (7). A “dual-hit hypothesis” was later formulated concerning the pathogenesis of idiopathic PD. This hypothesis describes a neurotropic pathogen which enters the nervous system through the nasal mucosa (via the olfactory pathways) and intestinal mucosa (via enteric plexuses and preganglionic vagal fibers), ultimately leading to a cascade of neurodegenerative events in the substantia nigra pars compacta (SNpc) (8).

This review highlights the association between important infectious pathogens and Parkinsonism, including symptoms of Parkinsonism following infectious etiologies, infectious contributions to neuroinflammation and neurodegenerative processes associated with Parkinsonism, and the epidemiologic correlation of infectious pathogens to idiopathic PD (Tables 1, 2).

**Table 1:** Clinical, histological, and structural features of Parkinsonism in infectious diseases.

**Species**	**Disorder**	**Clinical features**	**Histological/structural features**	**References**
Influenza A	Post-infectious encephalitis	EPS symptoms predominant (bradykinesia, tremor, mask-like features) No cognitive disturbances	Neuronal loss and neurofibrillary tangles in snpc Absence of lewy body deposition	(9, 10)
EBV	EBV encephalitis	Akinetic-rigid mutism, tremor, apraxia of eyelid opening	Progressive putaminal and caudate atrophy	(11–13)
VZV	Herpes Zoster	Cardinal symptoms of PD during follow-up, especially first 3 months after diagnosis		(14)
JEV	Japanese encephalitis	Varying severity of rigidity, hypokinesia, masking of the face Lower frequency of tremor Prominent hypophonia Most symptoms improve with time	Structural damage to the thalamus, basal ganglia, and brainstem observed in MRI findings of JE patients with parkinsonian features	(15, 16)
WNV	West Nile encephalitis	Tremor, myoclonus, rigidity, bradykinesia, and postural instability Transient PD features (resolve over time)	Bilateral, focal lesions in the basal ganglia, thalamus, and pons observed on MRI Increased level of a-syn in post-mortem analysis	(17, 18)
HIV	AIDS dementia complex	Bradykinesia, postural instability, gait abnormalities, hypomimemetic facies, and disorders of ocular motilit	Higher prevalence of a-syn in snpc Presence of HIV in inflammatory infiltrates and glial cells of basal ganglia Absence of lewy bodies deposition in ADC	(19–22)
	HIV Parkinsonism	Parkinsonism features similar to idiopathic PD Distinct characteristics include bilateral onset, rapid symptom progression, abnormal eye movements, earlier development of motor complications		

**Table 2:** Association of infectious pathogens in PD development and pathogenesis.

**Species**	**Associations with PD development**	**Indicated role in PD pathogenesis**	**References**
Influenza A	Risk of PD development in individuals who were previously infected with influenza virus not shown to be increased Inverse relationship between PD and influenza vaccinations has also been reported	Elevations in inflammatory cytokines leading to mitochondrial injury, development of oxidative stress, and neuronal apoptosis Direct contribution to transient dopaminergic neuronal loss in snpc: synergistic effect with MPTP, effect eliminated through the use influenza vaccinations or treatment with oseltamivir carboxylate Permanent activation of microglia: subsequent neuroinflammation	(23–33)
HSV-1	Elevated serological measure of HSV-exposure in PD patients correlated to disease severity	Molecular mimicry between HSV-1 and a-syn in the membranes of dopaminergic neurons of snpc: autoantibodies against HSV cross-react with a-syn epitope and promote a-syn aggregation	(34–36)
EBV	EBV seropositivity higher in PD patients than general population	Molecular mimicry between EBV and a-syn: anti-EBV latent membrane protein antibodies cross-react with a-syn and a-syn promote aggregation	(11, 37)
VZV	Increased risk of PD development with prior herpes zoster Childhood infections with varicella inversely related to PD	Overlapping mechanisms of neuroinflammation and immunological changes leading to neuronal death in both herpes zoster and PD	(14, 23)
HCV	Increased rate of PD development in patients with previous hepatitis C infection	Expression of HCV receptors on microvascular endothelial cells of the brain allow viral entry and CNS infection HCV upregulates chemokines leading to neuroinflammation, neuronal apoptosis, and dopaminergic toxicity HCV down-regulates TIMP-1 (astrocyte-derived factor known to promote neuronal survival during neurotoxicity)	(38–42)
JEV	Higher incidence of prior JEV infection among PD patients compared to the control	Damage to dopaminergic and norepinephrinergic systems Structural damage to the thalamus, basal ganglia, and brainstem observed in MRI findings of JE patients with parkinsonian features	(43, 44)
WNV		WNV-induced death of dopaminergic neurons	(45)
HIV	PD prevalence in persons living with HIV was similar to that of the general population Earlier onset of PD in HIV patients	Chronic neuroinflammation leading to basal ganglia dysfunction, altered blood-brain barrier permeability, and neurodegeneration Genetic associations °HIV exposure lead to dysregulated expression of DJ1 °Pathogenetic similarities between HIV-associated neurologic disorders and LRRK2	(21, 46–49)
*H. Pylori*	Increase prevalence of *H. Pylori* infection in PD patients compared to healthy controls *H. Pylori* infection associated with increased risk of subsequent PD in the general population PD patients with *H. Pylori* seropositivity display symptoms of worse motor severity Eradication of *H. Pylori* improves motor function in PD patients	Chronic inflammation and release of pro-inflammatory cytokines leading to BBB dysfunction, microglial activation, and neuronal injury Molecular mimicry between *H. Pylori* and proteins essential for normal neurological functions (NFIA, PDGFB, and EIFA3)	(34, 50–60)

## Viral Etiologies

### Influenza a Virus

The premise of a causative association between influenza virus and PD stems from the outbreak of encephalitic lethargica and postencephalitic Parkinsonism (PEP) which occurred in the aftermath of the 1918 influenza pandemic. Although the two events are temporally coincidental, influenza virus has not been confirmed as a direct causation to encephalitic lethargica and PEP. PEP is clinically and pathologically distinct from idiopathic PD. The overlapping clinical features include the classic extrapyramidal symptoms of bradykinesia, tremor, and “mask-like” features. However, patients affected with PEP do not exhibit cognitive disturbances such as aphasia and apraxia. Pathological evidence of neuronal loss and neurofibrillary tangles were similarly observed in the substantia nigra. Unlike in idiopathic PD, there is an absence of Lewy bodies deposition on histological samples from patients with PEP (9).

Although reports of PEP have become extremely rare in the last decade when compared to almost 50% of all diagnosed cases of Parkinsonism between 1925 and 1938, clinical research has suggested the role of influenza A virus in the processes of neuroinflammation and neurodegeneration contributing to the development of Parkinsonism. Transient neurological sequelae (including tremor and gait disturbance) have been reported in association with influenza infections, particularly within the first few weeks of diagnosis (10). A significant link between severe influenza and PD, as well as an inverse relationship between PD and influenza vaccinations has also been reported (23). Although the association of influenza and symptoms of Parkinsonism has been indicated, the risk of developing idiopathic PD in individuals who were previously infected with influenza virus was not shown to be increased (10).

The increasing risk of developing Parkinsonism is associated with increasing number of influenza attacks, suggesting that influenza-associated neuronal injury may be a cumulative inflammatory process (10). Individuals with susceptible genetic makeup, may suffer from immunologically mediated mitochondrial injury and development of neuronal oxidative stress subsequent to influenza-induced pyrexia and increased inflammatory cytokines, ultimately resulting to neuronal apoptosis. This is supported by findings of increased pro-inflammatory mediators, including interleukin 6 (IL-6) and tumor necrosis factor alpha (TNF-α), elevated levels of cytochrome C, a marker of mitochondrial injury, and reactive oxygen species (ROS) production in infected individuals, which point to the underlying immunological mechanisms in the pathophysiology of PEP (24–26).

On the other hand, a study on animal models found that the H5N1 influenza virus, upon its progression to the central nervous system (CNS) from the peripheral nervous system, is able to activate the innate immune response in the brain and cause the degeneration of dopaminergic neurons in the SNpc (27). Though this transient dopaminergic neuronal loss was found to be mostly restored within 90 days of infection, a long-lasting inflammatory response—permanent activation of microglia—persisted (28). The sustained activation of microglial cells was also reported after H1N1 infection, suggested to be a non-neurotropic virus, supporting the possibility that the virus may initiate inflammatory signals via direct microglia activation, contributing to disorders of protein aggregation, and neurodegeneration pathologies in the CNS (29). Synergistic effects of influenza and the parkinsonian toxin 1-methyl-4-phenyl-1,2,3,6-tetrahydropyridine (MPTP) have been observed in animal models infected with H1N1, in which the cumulative effects induced a greater loss of SNpc dopaminergic neurons than either insult alone. This loss of dopaminergic neurons is shown to be eliminated through the use of influenza vaccinations or treatment with oseltamivir carboxylate (30). These findings of synergistic effects from multiple insults supports the “multiple hit hypothesis,” where the combination of toxic stress and an inhibition of neuroprotective response lead to neuronal death (31, 32). Despite recent histologic evidence in animal models, unclear conclusions can be drawn from these findings as no significant neuropathological evidence of direct viral invasion in the CNS has been presented in infected individuals (33).

### Herpes Simplex Virus 1

The role of herpes simplex virus 1 (HSV-1) in the development and progression of neuropsychiatric disorders has been reported. Of particular interest is its pathological effect in provoking beta-amyloid deposition, tau phosphorylation, and demyelination, leading to cognitive deficits observed in neurodegenerative disorders including Alzheimer's disease and multiple sclerosis (61, 62). With regards to PD, the serological measure of exposure to HSV-1 amongst other common infectious pathogens such as CMV and EBV is shown to be elevated. The level of this infectious burden was additionally found to correlate to the severity of clinical symptoms and higher levels of serum inflammatory cytokines and alpha-synuclein (α-syn), supporting the role of infection in the etiology of PD (34).

The involvement of autoimmunity in PD's pathogenesis and the hypothesis that HSV-1 infections may lead to progression of the disease has been investigated. Recent findings indicate a mechanism of molecular mimicry between HSV-1 and α-syn in membranes of dopaminergic neurons of the SNpc. A difference in the level of autoantibodies recognizing HSV-1-Ul42_22−36_ was observed in PD patients compared to healthy controls. The antibodies were able cross-react with the homologous α-syn_100−114_ epitope, potentially promoting α-syn aggregation. These results suggest that HSV-1 may play a role in triggering an autoimmune response in PD, leading to dopaminergic neuronal destruction (35, 36).

### Epstein-Barr Virus

Statistical evidence suggests that Epstein-Barr virus (EBV) seropositivity in patients with PD was found to be higher than that of the general population. Rare incidences of Parkinsonism in EBV infection, specifically EBV encephalitis, comprises the clinical symptoms of akinetic-rigid mutism, tremor, and apraxia of eyelid opening. Structural brain changes including progressive putaminal and caudate atrophy were reported with one case showing direct acute neutropic effect of EBV on nigral dopaminergic cells (11–13). Similar to HSV-1, the evidence for molecular mimicry with α-syn, although currently speculative, has been indicated in EBV infection and PEP. Specifically, anti-EBV latent membrane protein antibodies targeting the critical repeat region cross reaction with the homologous epitope on the α-syn and induce its oligomerization (11, 37).

### Varicella Zoster Virus

Herpes zoster results from the reactivation of latent varicella-zoster virus (VZV) as a result of a decline in human cell-mediated immunity (63). A recent population-based cohort study in Taiwan found an increased risk of PD development among individuals with prior diagnosis of herpes zoster (age > 65 years) (14). Based on the overlapping mechanisms of neuroinflammation, immunological changes and resulting neuronal death in the two conditions, patients with herpes zoster may present with cardinal symptoms of PD during their follow-up period. These symptoms, particularly in the first 3 months after herpes zoster diagnosis, can lead to an earlier detection of PD (14).

This potential link was initially proposed by Ragozzino et al. (64), although the results were deemed equivocal due to limitations of the study (64). Findings reported that varicella, on the other hand, was not associated with PD (65). In fact, childhood infections with chicken pox were found to be inversely related to PD, suggesting a possible protective mechanism (23). This may be due to the “hygiene hypothesis,” in which immune challenges in early childhood are believed to support the development of a strong immune system later in life, though more evidence is necessary to develop a stronger connection (66).

### Hepatitis C

Hepatitis C virus (HCV) primarily infect hepatocytes, leading to progressive liver diseases. Its association with a number of CNS abnormalities including cognitive dysfunction, fatigue, and depression has also been well-documented (38). The development for HCV-associated neuropathology was described through the findings of Fletcher et al. in which the expression of functional HCV receptors (CD81, claudin-1, occluding, LDLR, scavenger receptor-B1) were found on microvascular endothelial cells of the brain, paving a pathway for HCV entry and replication into the CNS (39).

A recent meta-analysis demonstrated an increased risk of subsequent PD in patients with and hepatitis C (40). As there are no observed associations between autoimmune and chronic hepatitis to PD, it has been suggested that a specific aspect of viral hepatitis, rather than the general hepatic inflammatory process, contributes to these findings (41). HCV-induced upregulation of chemokines (including sICAM-1 and RANTES) in animal models which mediate mechanisms of neuroinflammation, neuronal apoptosis, and dopaminergic toxicity, was found to be similar to that of toxicity caused by 1-methyl-4-phenylpyridinium (MPP+) (42). In addition, HCV was shown to down-regulate TIMP-1, one of the neuroprotectants derived from astrocytes known to promote neuronal survival subsequent to toxic effects during neuroinflammation.

### Japanese Encephalitis Virus, West Nile Virus

Japanese encephalitis virus (JEV) and West Nile virus (WNV) are two important examples of zoonotic viral encephalitides. The clinical spectrum of these flaviviruses ranges from self-limiting flu-like illness to severe fatal meningoencephalitis, often with parkinsonian features (17, 67). Japanese encephalitis presents a wide spectrum of movement disorders including hypokinesia, tremor, rigidity, and dystonia. A transient form of parkinsonian syndrome, characterized by varying severity of rigidity, hypokinesia, masking of the face, lower frequency of tremor and prominent hypophonia, was observed in the acute stage of the illness after 1–4 weeks after the disease onset. Hypophonia was striking in most patients and was an important sequelae after the substantial regression of other manifestations in the subsequent months (15). Reports of Parkinsonism as a long-term sequela of JEV was also presented in patients 3–5 years after acute JE infection with associated lesions in the substantia nigra observed on MRI (16).

Burdwan, India has witnessed several outbreaks of JEV over the past two decades. Das et al. reported a higher incidence of prior JEV infection among PD patients within this region, compared to the control subject, suggesting the viral association to PD development (68). The analysis of JEV-induced Parkinsonism model rats showed decreased dopamine levels and neuropathological changes resembling those with idiopathic PD (43). Evaluation of cerebrospinal fluid in JEV-infected patients with movement disorders showed lower concentrations of norepinephrine, dopamine, and homovanillic acid, with respect to patients with non-JEV movement disorders. This observed decrease in catecholamine levels may be a result of damage to the dopaminergic and norepinephrinergic systems (44). Structural damage to the thalamus, basal ganglia, and brainstem observed in magnetic resonance imaging (MRI) findings of JE patients with parkinsonian features may contribute to this damage (44).

Features of Parkinsonism in acute WNV illness, including tremor myoclonus, rigidity, bradykinesia, and postural instability, are prominently reported and appear in most cases to be transient and resolve over time (18). These manifestations are uncommon among WNV-seronegative patients. Parkinsonian features were present in patients both in the presence and absence of MRI abnormalities showing bilateral, focal lesions in the basal ganglia, thalamus, and pons (17). Post-mortem analysis showed an increased level of α-syn in WNV-infected individuals. An increase in α-syn expression was also observed subsequent to WNV infection of primary neurons *in vitro*. The introduction of WNV into α-syn-knockout mice models were conducted, in which a 10-fold increase in viral production, increased neuronal injury, and a more rapid mortality was observed. This suggests the potential role of α-syn in inhibiting viral infection, rather than incurring CNS damage following viral infection. This data implies that the acute onset of parkinsonian features during WNV encephalitis is a result of the WNV-induced death of dopaminergic neurons (45).

### Human Immunodeficiency Virus

Parkinsonism is a common movement disorder in human immunodeficiency virus (HIV), occurring in up to 5% of infected individuals in the context of neuroleptic drugs exposure, cerebral opportunistic infections or AIDS dementia complex (ADC) (69). Parkinsonism may occur early in HIV infection reflecting viral infection within the basal ganglia or late in the disease course in combination with ADC. The basal ganglia and dopamine-rich brain regions are a vulnerable target to HIV, and Parkinsonism may develop as a result of underlying chronic neuroinflammation leading to basal ganglia dysfunction, altered blood-brain barrier (BBB) permeability, and neurodegeneration (19).

Bradykinesia, postural instability, gait abnormalities, hypomimemetic facies, and disorders of ocular motility, are common parkinsonian manifestations of ADC, a collective of neuropsychiatric complications in HIV (20–22). The clinical features of HIV Parkinsonism are similar to idiopathic PD. Several distinctions include bilateral onset, rapid symptom progression, abnormal eye movements, and earlier development of motor complications (69). The introduction of highly active antiretroviral therapy (HAART) has contributed to the evident decrease in AIDS-related Parkinsonism, while dopaminergic medication may also help to alleviate some symptoms of Parkinsonism and could be used in selected cases in which the benefits outweigh the medication's adverse effects (20, 70).

PD prevalence in persons living with HIV was similar to that of the general population. Earlier symptom onset (before the age of 60) was noted (21, 46). Studies of brain tissue from postmortem autopsies showed a higher prevalence of α-syn in the SNpc in individuals with HIV infection compared to healthy control samples. The presence of HIV preferentially in inflammatory infiltrates and glial cells of the basal ganglia including the substantia nigra were also observed (47). In ADC, however, typical pathological features in brains of patients with PD, such as Lewy bodies, have not yet been reported.

From a genetic viewpoint, HIV has been shown to affect the levels of PD-associated proteins, including DJ1 and Leucine-rich repeat kinase 2 (LRRK2). DJ1, a gene linked to early-onset PD, is a key regulator of dopamine transmission and ROS production in neuronal cells. Acute and chronic HIV exposure has been found to play a role in its dysregulated expression (48). LRRK2, a common genetic cause of familial and sporadic PD, shares pathogenetic and neurologic similarities with HIV-associated neurologic disorders. Pathologic LRRK2 activation was found to be an important mediator of neuroinflammation and neuronal damage in *in-vitro* and *in-vivo* models of HIV-associated neurologic disorders (49).

## Bacterial Etiology

### *Helicobacter pylori* Infection

*Helicobacter pylori* (*H. pylori*) is a bacterium on the luminal surface of the gastric epithelium that induces chronic inflammation of the underlying mucosa (71). It is found to be prevalent in the gastric mucosa of over half of the global human population (50, 72, 73). Ever since the initial observation of a high prevalence of gastric ulcers in addition to the gastrointestinal symptoms which preceded motor manifestations in PD patients, the link between *H. pylori* infection and the pathogenesis of PD has been extensively explored (51, 74). Stemming from the initial hypothesis of PD's origin within the gastrointestinal tract, the bidirectional interactions between the central and the enteric nervous system (namely, the gut-brain axis) is of particular interest (8). Research has supported the premise that *H. pylori* may play a role in the development of PD via several pathways. This centers around its involvement in neuronal damage, through the production of neurotoxic bacterial products and the disruption of the BBB. Another possible link is *H. pylori's* disruption of the intestinal microbiome, leading to altered inflammatory mediators that predispose to PD as part of the brain-gut axis interactions (75, 76).

Increased prevalence of *H. pylori* infection and higher titers of antibodies to *H. pylori* are observed among patients with PD compared to healthy controls. Accumulating evidence suggests that *H. pylori* infection is associated with increased risk of subsequent PD development in the general population. A positive correlation has been reported between worse motor severity and *H. pylori* seropositivity among patients (51–55). This is speculated to be a result of chronic inflammation exacerbating the neurodegenerative process or due to reduced absorption of anti-parkinsonian medication secondary to *H. pylori*-related gastroduodenitis. The improvement in clinical status, particularly symptoms of bradykinesia, subsequent to the eradication of *H. pylori* further supports a significant correlation (50, 53, 56–58).

*H. pylori* infection has been indicated to trigger neuroinflammation, neurotoxicity, and apoptosis related to the pathogenesis of PD. Chronic inflammation with *H. pylori* infection is associated with the substantial release of pro-inflammatory cytokines, which may lead to the disruption of the BBB, microglial activation and ultimately, neuronal injury (34, 59, 60). A subset of patients infected with *H. pylori* have been found to have elevated levels of autoantibodies against proteins essential for normal neurological functions (Nuclear factor 1 A-type (NFIA), Platelet Derived Growth Factor Subunit B (PDGFB), eukaryotic translation initiation factor 4A3 (EIFA3), suggesting a mechanism of molecular mimicry contributing to increased PD motor severity in *H. pylori* seropositive individuals (77).

### Other Bacterial Etiology

*Nocardia asteroides* is one of the bacterial influences that has been studied in PD pathoetiology over the past decades, initially stemming from the study by Kohbata et al. (78) which observed the development of motor abnormalities in animal models infected by a strain of *Nocardia asteroides*. This L-dopa-responsive movement disorder was found to be accompanied by neuronal inclusions which resembled lewy bodies (78). Nocardiae were reported to be able to propagate through neuroglia to neurons in mouse models. They are seen to multiply within the astroglia, through which they may invade midbrain neurons and induce neuronal loss and lewy body formation (79).

The association remains, however, inconclusive, as there is a lack of evidence for *Nocardia asteroides* in brain specimens from lewy body-containing disorders (80). Because spheroplasts found in the SNpc of PD patients have been shown to not be *Nocardia*, researchers have postulated that the bacterium known to form spheroplasts may alternatively be *Mycobacterium avium ss. Paratuberculosis* (MAP). MAP has been proposed to be the “unidentified enteric pathogen” that triggers α-syn aggregation (81).

Recent research has explored the relationship between gut dysbiosis and the onset and aggravation of PD. The interaction between intestinal microbiota and the autonomic and CNS has been indicated via diverse pathways including the enteric nervous system and the vagal nerve (82–84). In a recent study, *Proteus mirabilis* (commonly increased in the gut microbiota of PD mouse models) has been shown to directly induce PD-related pathological changes and motor deficits. This includes the induction of dopaminergic neuronal damage and inflammation within the substantia nigra and the striatum, and the stimulation of α-syn aggregation in the brains and colons of PD mice (85). Lipopolysaccharide (LPS), a virulence factor of *P. mirabilis*, has been implicated in these pathological changes. Increased intestinal permeability leading to a greater exposure of intestinal neuronal tissue to pro-inflammatory products have been suggested to result in oxidative stress and neuronal pathological α-syn aggregates (85, 86).

A number of bacterial infections have been explored in association with PD, including *Chlamydia pneumoniae* (34, 87), *Bordetella pertussis* (65, 88), *Streptococcus pyogenes* (65, 89), and *Borrelia burgdoferi* (34). The varying results amongst epidemiological and laboratory studies are primarily suggestive, as there is inadequate evidence to support their role in PD development. The relationship between these bacterial etiologies and PD requires more comprehensive studies in the future.

## Pathomechanisms of Post-Infectious Parkinsonism

### Neuroinflammation

Neuroinflammation is a common feature of neurodegenerative disorders of the CNS, characterized by augmented numbers of activated and primed microglia, increased level of inflammatory cytokines and decreased levels of anti-inflammatory molecules. Neuroinflammation might initiate from the periphery and relative data suggest that peripheral conditions through the disrupted BBB notably influence various pathologic processes in the brain (60). Subsequent to its access to the CNS through the systemic circulation, the olfactory pathways, and the gastrointestinal tract, infectious pathogens may induce neuroinflammation through the induction of pro-inflammatory cytokines in microglia (27, 29).

The activation of microglia and release of inflammatory factors promote damage to DA neurons. Infectious pathogens have been shown to be associated with the release of large amounts of proinflammatory mediators, including TNF-α, IL-6, and IL-1β which may lead to disruption of the BBB, microglial activation and, ultimately, neuronal injury and death (90). Such relationships between the innate autoimmune response and infectious pathogens has also been investigated by other literature such as Caggiu et al. (91).

**Figure 1:**
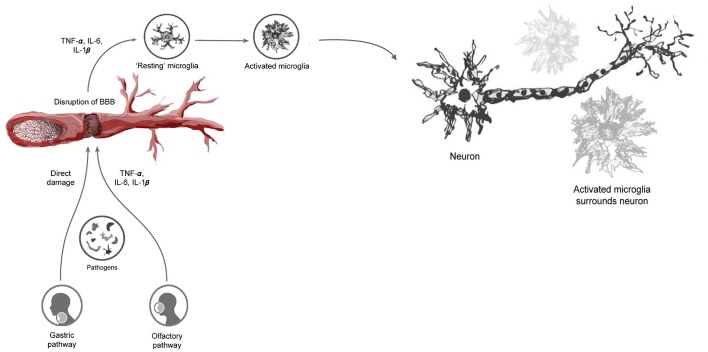
Infectious pathogens inciting the neuroinflammatory process and subsequent blood-brain barrier disturbance through the release of pro-inflammatory cytokines. This ultimately leads to the activation of microglia and subsequent clustering around neuronal cells, resulting in neuronal damage.

This mechanism of microglial activation and hypercytokinemia can cause further activation and clustering of microglia around DA neurons, leading to a continuous cycle of chronic inflammation and neuronal damage. Chronic activation of microglia and release of cytokines have been shown to cause extensive damage to the dopaminergic neurons in the SNpc, contributing significantly to neuronal death in PD (92, 93) (Figure 1).

From another view point, viral agents through their replication and subsequent cell lysis may result in direct neuronal damage (30, 94). Similarly, vascular inflammation due to hypercytokinemia can lead to apoptosis of endothelial cells and loss of vascular integrity, which result in increased permeability of cerebral vessels and cerebral edema and ultimately neuronal damage.

### Role of Alpha-Synuclein in the Immune System

A-syn is a presynaptic neuronal protein involved in neurotransmitter release that is thought to play a significant role in the initiation and progression of PD. A-syn is also a major constituent of Lewy bodies and Lewy neurites, which are well-known pathological hallmarks of the disease. Recent studies suggest that oligomers or protofibrils of α-syn, rather than the fibrils, are responsible for the toxic effects causing neurodegeneration (95–97). Oligomerized α-syn is hypothesized to have toxic effects on synaptic transmission (98), cause membrane disruption (99), impairment of protein degradation, as well as impairment of several organelles including the mitochondria and endoplasmic reticulum (100, 101). The ability of α-syn to cause membrane disruption and organelle impairment has also been hypothesized to give the protein antimicrobial properties. This was demonstrated in a study by Park et al. in which recombinant a-syn proteins were used in antimicrobial assays against various bacterial and fungal strains (102). This antimicrobial effect could suggest a protective mechanism of the post-infectious increase in α-syn oligomers.

**Figure 2:**
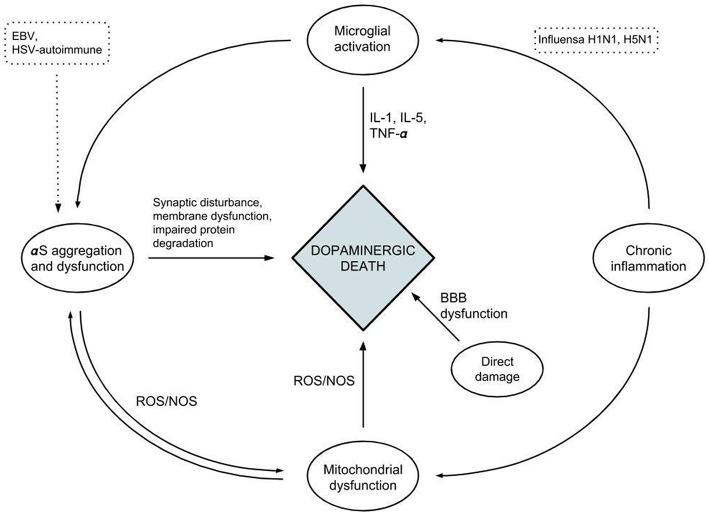
A diagram depicting the relationships between the different factors affecting neuroinflammation and neuronal cell death.

Bidirectional relationships have been proposed between α-syn and microglia through the activation and release of inflammatory mediators, potentiating each other and causing dopaminergic neuronal loss and neurodegeneration (103). A similar relationship is also seen between α-syn and mitochondria, in which α-syn oligomers cause disruption of mitochondrial function and the resultant oxidative stress in turn furthers the oligomerization of α-syn. It has been proposed that aggregated α-syn may function as a messenger molecule for immune responses against infectious pathogens by inducing microglial activation. This is similar to the proinflammatory cytokines' incitation of antimicrobial responses within the intestinal epithelium (83, 104–106). Together with its antimicrobial and antifungal effect, this supports a potential immunoprotective role of α-syn. Viral agents have been shown through these mechanisms of molecular mimicry to induce the aggregation and oligomerization of α-syn, leading to deposition in neuronal cells and further damage (35, 36) (Figure 2).

## Clinical Implications

Currently, the diagnosis of PD relies on the clinical presence of the disease cardinal features, including resting tremor, rigidity, bradykinesia, and loss of postural reflexes. The efficacy of this diagnostic method is limited during the early stages of the disease, permitting PD progression before any therapeutic intervention. The positive correlation of PD development amongst patients with underlying chronic infection suggests the benefit of PD screening before progressive symptom onset. Neurological examinations may be beneficial to detect the earlier stages of PD in, for example, anti-HCV positive groups, which would be an important clinical implication for high endemic HCV areas. Conversely, PD patients may benefit from an evaluation for underlying infection as clinical improvement of motor symptoms have been shown subsequent to the eradication of disease, for example as in chronic *H. pylori* infection (50, 53, 56–58). Whether the clinical deterioration observed in these cases is a result of chronic inflammation exacerbating the neurodegenerative process or due to reduced absorption of anti-parkinsonian medication secondary to *H. pylori*-related infection is a topic for further investigation. On the other hand, anti-parkinsonian drugs may have possible therapeutic antiviral potential. For example, Parkinsonian drugs (L-dopa, Isatin, Amantadine) have shown *in-vitro* to significantly inhibit WNV multiplication as well as reduced viral RNA levels (Amantidine) (107).

Another significant limitation in diagnosis is that the neurodegenerative process associated with PD precedes the onset of clinical symptoms, in which up to 70% of neurons in the SNpc are lost before the appearance of motor features (108, 109). A-syn oligomers could serve as a biomarker that would allow the identification of at-risk individuals before clinical diagnosis. Detection of α-syn oligomers may additionally be useful in monitoring disease progression and response to treatments. Emerging information on the pathomechanism of diseases such as loss of BBB integrity may also lead to formulating strategies to protect BBB and to prevent and treat PD and other BBB-related neurodegenerative disorders (110, 111).

## Conclusions and Future Perspective

Accumulating evidence in recent years implicates the role of infectious etiologies in neuronal pathways leading to the development of Parkinsonism and PD. Studies have demonstrated the independent association between specific pathogens and PD. The synergistic effect of infectious pathogens in inducing neuroinflammation leading to the PD development have also been observed. Neuroinflammation may not only play a part in the precipitation of PD but may also be a sustaining factor in its progression. However, the pathomechanism of dopaminergic neuronal loss in infection-induced Parkinsonism cannot explain the pathogenesis of idiopathic PD. The conclusion that all cases of PD are associated with increased inflammation and underlying chronic infection cannot be established. This is due multiple factors, including the consideration that not all PD patients have consistent evidence of inflammatory cytokine dysregulation and that the state of increased inflammation in individuals do not always lead to PD development. Parkinsonism development may be part of a response to an underlying immune system dysregulation in individuals with genetic predisposition to the disease.

Further research is necessary to examine the involvement and extent to which pathogens and inflammatory cytokines play in the pathomechanism of PD. Confounding factors, including the roles of genetics and exposure to environmental insults, have to be taken into account in further investigation. Studies should explore the possibility of a subtype of PD that is characteristically associated with pro/anti-inflammatory cytokines and the reason why certain individuals develop PD in the context of elevated inflammatory markers. Through further understanding these mechanisms, we may be able to classify variants of PD, use biological markers to aid in the diagnosis and prediction of treatment response and adjust treatment for an improved prognosis for patients.

## Author Contributions

NL: conception and design of the study, acquisition and analysis of data, drafting the manuscript and figures, project administration, supervision, validation. PI: conception and design of the study, supervision, validation. MH: acquisition and analysis of data, drafting the manuscript and figures. DK: drafting the manuscript and figures. WK: supervision, validation.

### Conflict of Interest Statement

The authors declare that the research was conducted in the absence of any commercial or financial relationships that could be construed as a potential conflict of interest. The reviewer PB and handling Editor declared their shared affiliation at the time of review.
